# The complete genome sequence and analysis of a plasmid-bearing myxobacterial strain *Myxococcus fulvus* 124B02 (M 206081)

**DOI:** 10.1186/s40793-015-0121-y

**Published:** 2016-01-04

**Authors:** Xiao-jing Chen, Kui Han, Jing Feng, Li Zhuo, Ya-jie Li, Yue-zhong Li

**Affiliations:** State Key Laboratory of Microbial Technology, School of Life Science, Shandong University, Jinan, 250100 China

**Keywords:** *Myxococcus fulvus* 124B02, Complete genome sequence, Endogenous plasmid, Autonomously replicate, Genome expansion

## Abstract

Myxobacteria, phylogenetically located in the delta division of the *Proteobacteria*, are well known for characterized social behaviors and large genomes of more than 9 Mb in size. *Myxococcus fulvus* is a typical species of the genus *Myxococcus* in the family *Myxococcaceae. M. fulvus* 124B02, originally isolated from a soil sample collected in Northeast China, is the one and only presently known myxobacterial strain that harbors an endogenous autonomously replicating plasmid, named pMF1. The endogenous plasmid is of importance for understanding the genome evolution of myxobacteria, as well as for the development of genetic engineering tools in myxobacteria. Here we describe the complete genome sequence of this organism. *M. fulvus* 124B02 consists of a circular chromosome with a total length of 11,048,835 bp and a circular plasmid of 18,634 bp. Comparative genomic analyses suggest that pMF1 has a longstanding sustention within myxobacteria, and probably contributes to the genome expansion of myxobacteria.

**Electronic supplementary material:**

The online version of this article (doi:10.1186/s40793-015-0121-y) contains supplementary material, which is available to authorized users.

## Introduction

The gliding Gram-negative myxobacteria are characterized by complex social behaviors, *e.g.* cells moving on solid surfaces in swarms, preying on other microorganisms in a ‘wolf-like’ pattern, and, when nutrients are depleted, developing into myxospores embodied in fruiting bodies [1, 2]. In addition, myxobacteria are able to produce various secondary metabolites and macromolecule degradation enzymes, not only having potential in applications but also probably working as ecological weapons against other living microorganisms [3–5]. Myxobacteria possess large genomes. For instance, the genomes of *Myxococcus xanthus* DK1622 and the halotolerant *M. fulvus* HW-1 are 9.14 Mb [6] and 9.03 Mb [7] in size, while the genomes of *Sorangium cellulosum* even reach to 13.03 Mb in strain So ce56 [8] and 14.78 Mb in strain So0157-2 [9], respectively. The So0157-2 genome is still the largest one reported in prokaryotes.

Extrachromosomal autonomously replicating genetic materials are normally absent from myxobacterial cells. Up to now, pMF1, originally discovered from *M. fulvus* 124B02 [10], is still the one and only endogenous plasmid that is able to replicate autonomously in myxobacterial cells. Genome sequencing of *M. fulvus* 124B02 is thus meaningful for understanding the evolution of myxobacterial genomes, and providing clues for the presence of pMF1 in strain 124B02. Here we report the complete genome sequence and analyses of *M. fulvus* 124B02.

## Organism information

### Classification and features

**Fig. 1:**
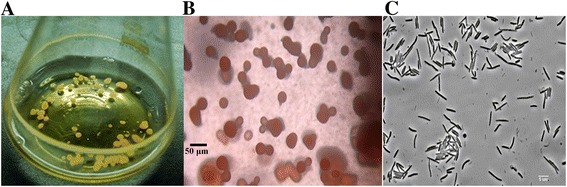
Morphological characteristics of *M. fulvus* 124B02. **a** Spherical clumps of strain 124B02 cells cultivated in liquid CYE medium. **b** Fruiting bodies formed on the TPM development medium. The pictures were taken after six days of incubation under a stereoscopic microscope, Bar = 50 μm. **c** Vegetative cells, Bar=5 μm

Strain 124B02 was isolated from a soil sample collected in Northeast China [11]. Vegetative cells of the strain are slender rods with tapering ends, 0.6-0.8 × 4–8 μm. The fruiting bodies are spherical or slightly pear-shaped with a diameter of 50–250 μm and a yellow red color. The strain did not grow expansively, but into membranaceous clumps on CYE solid plates. When grown in liquid CYE medium, the cells grew into spherical clumps. Figure 1 shows morphological characteristics of *M. fulvus* 124B02. The optimal growth pH for strain 124B02 is in the range of 6.8–7.6, and the optimal growth temperature ranges between 26 °C and 32 °C. The predominant fatty acids of *M.fulvus* 124B02 cells were determined as *iso*-C_15:0_ (33.18 %), C_16:1_ ω5c (20.19 %), *iso*-C_14:0_ 3-OH (6.27 %), C_16:0_ (5.79 %) and C_14:0_ (5.65 %). 2-hydroxy and 3-hydroxy fatty acids are the major hydroxyl fatty acid components of strain 124B02.

**Fig. 2:**
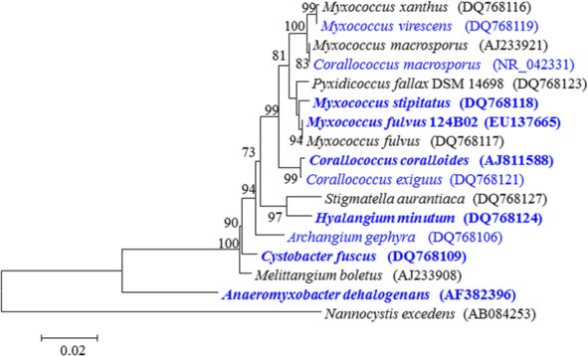
Phylogenetic tree showing the position of *M. fulvus* 124B02 within the *Cystobacterineae*. The tree was inferred from 1,445 aligned bases [35, 36] of the 16S rRNA gene sequence under the maximum likelihood criterion [37] and rooted with *Nannocystis excedens*. The branches are scaled in terms of the expected number of substitutions per site. Numbers above branches are the supporting values from 1,000 bootstrap replicates. Lineages with type strain genome sequencing projects registered in GOLD [38] are shown in blue and published genomes in bold

Figure 2 is a phylogenetic tree of the 16S rRNA gene sequences showing the location of *M. fulvus* 124B02 in the *Cystobacterineae* suborder of myxobacteria (the GenBank accession number of the 16S rRNA gene sequence of strain 124B02 is EU137665). All three 16S rRNA gene copies in the genome of strain 124B02 are identical, but differ by two nucleotides from the previously published 16S rRNA sequence generated from *M.fulvus* 124B02 (EU137665). According to the morphological and phylogenetic characteristics, *M. fulvus* 124B02 was determined as a typical strain of *Myxococcus fulvus* (Table 1 shows the classification and general features of the strain).

**Table 1:** Classification and general features of *M. fulvus* 124B02 according to the MIGS recommendations [39]

MIGS ID	Property	Term	Evidence code^a^
	Classification	Domain *Bacteria*	TAS[40]
		Phylum *Proteobacteria*	TAS[41]
		Class *Deltaproteobacteria*	TAS[42]
		Order *Myxococcales*	TAS[43]
		Suborder *Cystobacterineae*	TAS[44]
		Family *Myxococcaceae*	TAS[45]
		Genus *Myxococcus*	TAS[46]
		Species *Myxococcus fulvus*	TAS[47, 48]
		Strain 124B02	TAS[10]
	Gram strain	Negative	TAS[44]
	Cell shape	Rod	TAS[1]
	Motility	Motile	TAS[1]
	Sporulation	Myxospore	TAS[1]
	Temperature range	Mesophile, 25–35 °C	TAS[44]
	Optimum temperature	26–32 °C	TAS[44]
	pH range; Optimum	6.4–8.8; 6.8–7.6	TAS[47]
	Carbon source	Macromolecules such as proteins	TAS[44]
MIGS-6	Habitat	Soil	IDA
MIGS-6.3	Salinity	Non-halophile	NAS
MIGS-22	Oxygen requirement	Aerobic	TAS[1]
MIGS-15	Biotic relation	Free-living	NAS
MIGS-14	Pathogenicity	Non-pathogen	NAS
	Biosafety level	1	TAS[49]
	Isolation	Soil	IDA
MIGS-4	Geographic location	Changchun, China	IDA
MIGS-5	Sample Collection	1999	IDA
MIGS-4.1	Latitude	not reported	
MIGS-4.2	Longitude	not reported	
MIGS-4.3	Depth	not reported	
MIGS-4.4	Altitude	not reported	

^a^Evidence codes – *TAS* traceable author statement, *i.e.* the direct report in the literature, *NAS* non-traceable author statement, *i.e.* not directly observed for the living, isolated sample, but based on a generally accepted property for the species, or anecdotal evidence. These evidence codes are from the Gene Ontology project [19]

## Genome sequencing information

### Genome project history

This organism was selected for sequencing because of its evolutionary significance as the only presently known myxobacterial strain bearing an endogenous plasmid. The genome project of *M. fulvus* 124B02 was deposited in the Genome Online Database and the complete genome sequence of strain 124B02 was deposited in GenBank under the accession number of CP006003. A summary of the project information is shown in Table 2.

**Table 2:** Genome sequencing project information

MIGS ID	Property	Term
MIGS-31	Finishing quality	Finished
MIGS-28	Libraries used	Three genomic libraries: one 454 single read library,one 454 pair end library (3 kb), one Illumina pair end library (350)
MIGS-29	Sequencing platforms	Roche 454 GS FLX, Illumina GAII
MIGS-31.2	Fold coverage	25.8 × pyrosequence; 86.7 × Illumina
MIGS-30	Assemblers	Newbler assembler V2.3, Phrap, Velvet
MIGS-32	Gene calling method	GeneBank
	Locus Tag	MFUL124B02
	GenBank ID	CP006003
	GenBank Date of Release	May 6, 2015
	GOLD ID	Gp0043396
	BIOPROJECT	PRJNA203240
MIGS-13	Source material identifier	M 206081
	Project relevance	The host of plasmid pMF1

### Growth conditions and genomic DNA preparation

*M. fulvus* 124B02 was cultivated in the CTT growth medium containing 1 % casitone, 10 mM Tris–HCl, 1 mM KH_2_PO_4_-K_2_HPO_4_, 8 mM MgSO_4_, pH 7.6. The cells were harvested by centrifugation after five days of incubation at 30 °C. DNA was extracted from the cell mass using the methods described previously [12] with slight modifications. Briefly, approximately 50–100 mg cell pellets were suspended in 500 μl TE buffer, containing 25 mM Tris–HCl (pH 8.0), 25 mM EDTA, and 2 mg/ml lysozyme. The mixture was incubated at 37 °C for 1 h with periodic gentle inversion for cell lysis. Then, 2.5 μl proteinase K was added to a final concentration of 100 μg/ml, and the mixture was incubated at 37 °C for additional 1 h. The total protein was removed with Tris-saturated phenol-chloroform-isoamyl alcohol (25:24:1, pH 8.0). To precipitate DNA, 0.1 volume of 3 M sodium acetate (pH 5.3) and the same volume of isopropyl alcohol were added to the final supernatant. The DNA pellet was washed with 70 % ethanol twice, air-dried, and dissolved in 50 μl ddH_2_O.

### Genome sequencing and assembly

Genome sequencing and assembly were performed in Shanghai Majorbio Bio-Pharm Technology Co., Ltd. The genome was sequenced with a combination of the Roche 454 GS FLX and Illumina GAII sequencing platforms. The 454 pyrosequencing reads, containing 285.8 Mb draft data, were firstly assembled using the Newbler assembler V2.3, producing 51 contigs in 23 scaffolds. This initial assembly was converted into a phrap assembly by making fake reads from the consensus, to collect the read pairs in the 454 paired end library. The clean data from Illumina GAII sequencing were assembled with Velvet assembler and the consensus sequences were shredded into 800-bp overlapped fake reads, which were assembled with the 454 draft data. In total, the combination of the Illumina and 454 sequencing platforms produced 112.5× coverage of the genome. The final assembly contained 738,315 pyro sequences and 12,776,900 Illumina reads. After the shotgun stage, reads were assembled with parallel phrap (High Performance Software, LLC). Then the Phred/Phrap/Consed software package [13–15] was used for quality assessment. Possible misassembles were corrected by sequencing the cloned bridging PCR fragments. We designed primers for the amplification of 76 gap regions to close gaps and to improve the quality of the finished genome. Gaps between contigs were closed by editing in Consed, PCR amplification and 3730 sequencing. The wrong bases were corrected by comparing with Illumina GAII data after the genome cyclization, using BWA (0.7.3a) [16] and samtools (0.1.19) [17]. The error rate of the completed genome sequence is less than 1 bp in 100,000 bp.

### Genome annotation

The genome was annotated automatically in GenBank. In addition, we predicted Cluster Regularly Interspaced Short Palindromic Repeats (CRISPRS) with PILER-CR [18]. We analyzed the predicted protein sequences against the National Center for Biotechnology Information (NCBI) non-redundant database, Gene Ontology [19], KEGG [20], and COG [21] databases for functional annotation. The results were summarized with the InterProScan [22] software. To analyze the COG annotation, hits with an E-value < = 1e-5 were first retained. Then, only the best hit was selected for each protein. Signal peptides and transmembrane helices of all annotated proteins were predicted using SignalP 4.1 Sever [23] and TMHMM Sever v. 2.0 respectively.

## Genome properties

**Fig. 3:**
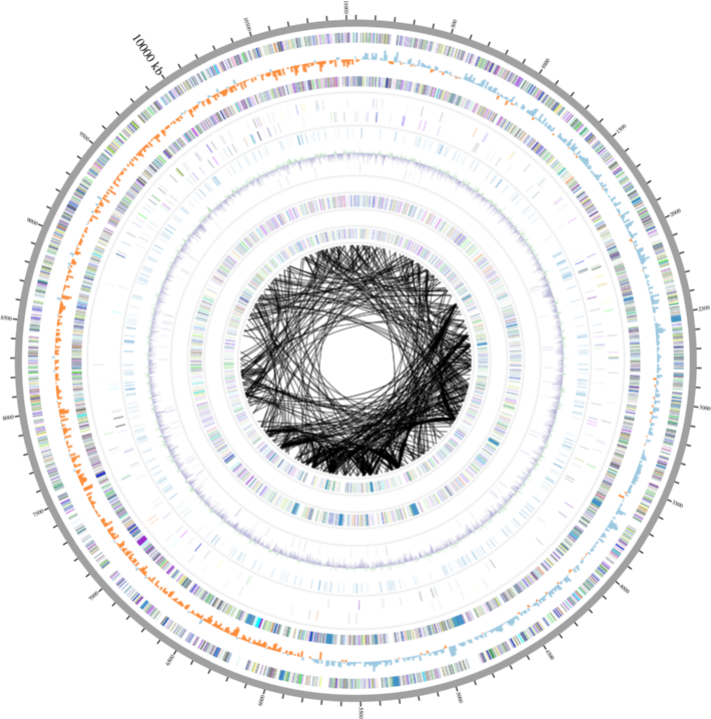
Schematic map of the genome. From outside: Circle 1, genome positions in kb (started from *dnaA*); Circles 2 and 4, predicted protein coding sequences (CDSs) on the forward (*outer wheel*) and the reverse (*inner wheel*) strands, colored according to COG classification; Circle 3, GC skew plot; The positive GC skew value (*blue*) corresponds to leading strand and negative GC value (*orange*) correspond to lagging strand. Circles 5 and 6, expanded genes in *M. fulvus* 124B02, compared with *M. stipitatus* DSM 14675; Circles 7, putative virus and prophage-derived CDSs; Circle 8, GC content showing deviations from the average (69.96 %); Circle 9 and 10, putative plasmid-derived CDSs (leading strand, 1,879 CDSs; lagging strand, 2,046 CDSs); Core circle, paralogous CDSs

The genome statistics are provided in Table 3, Table 4 and Fig. 3*.**M. fulvus* 124B02 consists of a circular chromosome with a total length of 11,048,835 bp and a circular plasmid of 18,634 bp. The G+C contents of the chromosome and the plasmid are 69.96 % and 68.7 %, respectively. There were 8,515 predicted coding sequences (CDSs) in the genome, including 9 rRNAs and 80 tRNAs. The protein coding sequences occupied 86.13 % of the whole genome sequence. The majority of the protein-coding genes (5,042, 58.24 % of the total) were assigned putative functions in categories of orthologous group (COG), while the remaining ones were annotated as hypothetical proteins. The distribution of genes in COGs functional categories is presented in Table 5.

**Table 3:** Information of *M. fulvus* 124B02 genome and the endogenous plasmid

Label	Size (bp)	Topology	INSDC identifier	RefSeq ID
Chromosome	11,048,835	Circular	CP006003	NZ_CP006003.1
Plasmid pMF1	18,634	Circular	EU137666.1	NC_010372.1

**Table 4:** Statistics of *M. fulvus* 124B02 genome

Attribute	Value	% of total^a^
Genome size (bp)	11,048,835	100.00
DNA coding (bp)	9,516,537	86.13
DNA G + C content (bp)	7,730,063	69.96
DNA scaffolds	0	
Total genes	8,658	100.00
Protein-coding genes	8,515	98.58
RNA genes	89	1.03
Pseudogenes	54	0.62
Genes in internal clusters	505	5.83
Genes with function prediction	4,749	54.85
Genes assigned to COGs	5,042	58.24
Genes with Pfam domain	5,929	68.48
Genes with signal peptides	984	11.37
Genes with transmembrances	1,605	
CRISPR repeats	1	

^a^The total is based on either the size of the genome in base pairs or the total number of protein coding genes in the annotated genome

**Table 5:** The genes of *M. fulvus* 124B02 genome in COG functional categories

Code	Value	%^a^	Description
J	203	2.38	Translation, ribosomal structure and biogenesis
A	5	0.06	RNA processing and modification
K	416	4.89	Transcription
L	205	2.41	Replication, recombination and repair
B	3	0.04	Chromatin structure and dynamics
D	44	0.52	Cell cycle control, cell division, chromosome partitioning
Y	-	-	Nuclear structure
V	129	1.51	Defense mechanisms
T	415	4.87	Signal transduction mechanisms
M	329	3.86	Cell wall/membrane/envelope biogenesis
N	117	1.37	Cell motility
Z	-	-	Cytoskeleton
W	-	-	Extracellular structures
U	65	0.76	Intracellular trafficking, secretion, and vesicular transport
O	235	2.76	Posttranslational modification, protein turnover, chaperones
C	227	2.67	Energy production and conversion
G	205	2.41	Carbohydrate transport and metabolism
E	342	4.02	Amino acid transport and metabolism
F	94	1.10	Nucleotide transport and metabolism
H	148	1.74	Coenzyme transport and metabolism
I	283	3.32	Lipid transport and metabolism
P	191	2.24	Inorganic ion transport and metabolism
Q	177	2.08	Secondary metabolites biosynthesis, transport and catabolism
R	767	9.01	General function prediction only
S	442	5.19	Function unknown
-	3,473	40.79	Not in COGs

^a^The percentage is based on the total number of protein coding genes in *M. fulvus* 124B02 genome

## Insights from the genome sequence

**Fig. 4:**
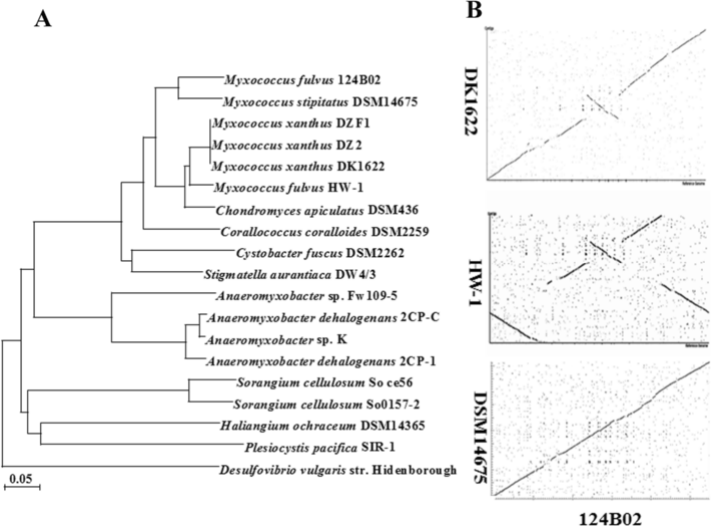
Phylogenomic analyses of *M. fulvus* 124B02. **a** A whole-genome phylogenomic tree of *M. fulvus* 124B02 and other sequenced myxobacterial strains using *Co-phylog* [50]. **b** The schematic maps of whole-genome synteny using blastn in NCBI between *M. fulvus* 124B02 and the other three sequenced *Myxococcus* strains, *i.e. M. xanthus* DK1622, *M. fulvus* HW-1 and *M. stipitatus* DSM 14675. Genomic inversions were observed in *M. fulvus* 124B02 *vs. M. xanthus* DK1622 and *M. fulvus* 124B02 *vs. M. fulvus* HW-1

Until now, 22 myxobacterial genomes have been released in NCBI database. Except for the anaerobic myxobacteria, whose genomes are approximately 5 Mb, all the aerobic myxobacteria have rather large genomes, ranging from 9.03 Mb of *M. fulvus* HW-1 to 14.78 Mb of *S. cellulosum* So0157-2. Compared with the other sequenced *Myxococcus* genomes, *i.e.* 9.14 Mb of *M. xanthus* DK1622 [6], 9.03 Mb of *M. fulvus* HW-1 [7], and 10.35 Mb of *M. stipitatus*DSM 14675 [24], the genome of *M. fulvus* 124B02 is rather large. It is known that horizontal gene transfer (HGT) [25, 26] and intra-chromosomal gene duplication (IGD) [27, 28] are two major contributors for the expansion of most prokaryotic genomes. BLASTP searching against the other three sequenced *Myxococcus* genomes revealed 576 strain-specific duplications in the strain 124B02 genome (the core circle in Fig. 3), accounting for 6.7 % of the total CDSs. The exogenous genetic materials may be introduced into bacterial genomes via plasmids, prophages, virus, integrative conjugative elements, insertion sequence elements or other unclassified elements [29]. Of the total 8,492 CDSs in *M. fulvus* 124B02 genome, 3926 (46.2 %) were probably derived from plasmids (circles 9 & 10 in Fig. 3), which is similar to that in other myxobacteria [6, 9]. We conducted an all-blast-all analysis using BLASTP program with an E-value cutoff of 1e-5, and the results were transferred into OrthoMCL package to extract the paralogous and orthologous proteins. Interestingly, the phylogenomic analysis indicated that *M.fulvus* 124B02 is closer to *M. stipitatus*DSM 14675, rather than *M. xanthus* DK1622 or *M. fulvus* HW-1 (Fig. 4a), which was also supported by the genome synteny analysis (Fig. 4b). We found that the major differences between *M. fulvus* 124B02 and the other three *Myxococcus* strains were those protein sequences for the metabolism and environment adaption processes [Additional file 1: Table S1, Additional file 2: Table S2, Additional file 3: Table S3] and of those strain-specific genes. For example, according to the COG catalog, the major differences between *M. fulvus* 124B02 and *M. stipitatus*DSM 14675 were in the families of lipid transport and metabolism (*p*-value is 0.0076, Fisher’s exact test, two-tailed test), transcription (*p*-value is 0.0097, Fisher’s exact test, two-tailed test), secondary metabolites biosynthesis, transport and catabolism (*p*-value is 0.0015, Fisher’s exact test, two-tailed test) and replication, recombination and repair (*p*-value is 0.0251, Fisher’s exact test, two-tailed test). *M. fulvus* 124B02 had approximately 1,230 kb strain-specific fragments, which scattered throughout the whole genome (circles 5 & 6 in Fig. 3). Additional file 4: Table S4 lists the strain specific genes of replication, recombination and repair family, of which the number of *M.fulvus* 124B02 is less than *M.stipitatus*DSM 14675.

**Fig. 5:**
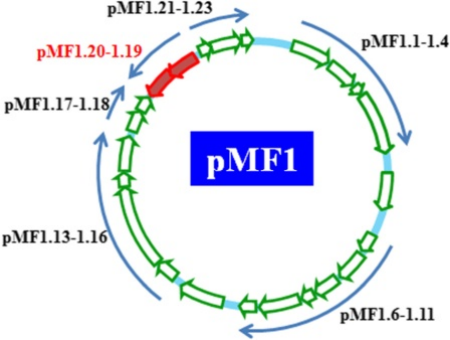
The organization of genes in pMF1. Except for *pMF1.19* and *pMF1.20* on the lagging strand, the other genes were located on the leading strand. A blue arrow means an operon

**Fig. 6:**
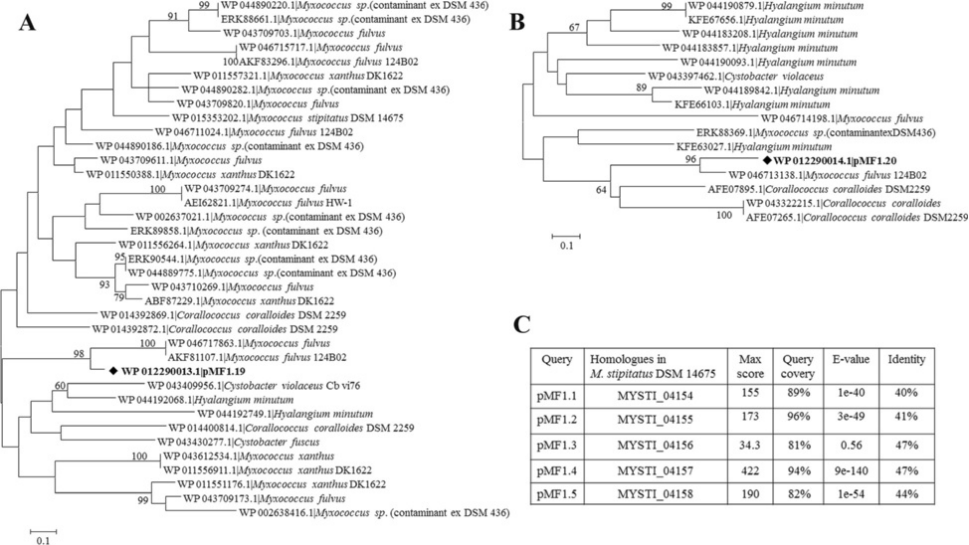
Homologues of pMF1 ORFs in myxobacterial genomes. **a & b** are the unrooted phylogenetic trees of the pMF1.19 and pMF1.20 proteins and their homologues, respectively. **c** is the summary of the homologues of pMF1.1-1.5 in *M. stipitatus* DSM 14675. The homologous proteins that have identity >= 40 %, E. value < 1×10^−5^ for pMF1.19, and identity >= 40 %, E. value < 1×10^−5^ for pMF1.20. The phylogenetic trees were constructed using maximum likelihood program with the Poisson correction distance model of MEGA5 [51]. The bootstrapping supports for the interior branch length of the trees were from 1,000 replicates

pMF1 is a low copy number plasmid, containing 23 predicted ORFs [10]. The plasmid has no obvious beneficial genes for persistence in host, such as the genes encoding for antibiotic resistance, virulence, or growth phenotypes. All the predicted genes in pMF1 are of unknown functions, except the replication system (*pMF1.13-pMF1.16*) and the partitioning system (*pMF1.21-pMF1.23*), both of which were determined by narrowing-down of sequence fragments [10, 30, 31]. While the *pMF1.19* and *pMF1.20* genes were on the lagging strand, the others were located on the leading strand (Fig. 5). Interestingly, BLASTP searching against the GenBank database showed that pMF1.19 and pMF1.20 had multiple homologues, mostly in pairs, in different myxobacterial genomes, except that of *Anaeromyxobacter*. For example, there were at least ten homologues of pMF1.19 and nine of pMF1.20 in *M. xanthus* DK1622. The predicted protein products of these two genes contained the conserved Pfam09535 and Pfam09533 domains, but lack significant sequence similarities to any known protein families. These function-unknown homologues exist in myxobacteria only. The identities of these homologues ranged from 31 % (WP_011550397, *M. xanthus* DK1622) to 74 % (MFUL124B02_18095 of *M. fulvus* 124B02) for pMF1.19 and from 30 % (WP_011550387, *M.xanthus* DK1622) to 88.0 % (MFUL124B02_18100 of *M. fulvus* 124B02) for pMF1.20 (Fig. 6a and b are phylogenetic trees of the pMF1.19 and pMF1.20 homologues, respectively). The homologues with highest similarities to pMF1.19 and pMF1.20 are both in *M. fulvus* 124B02 host genome, which suggested that pMF1 was more closely related with this strain than other myxobacteria. The homologues of pMF1.19 and pMF1.20 in the genome of *M. fulvus* 124B02 are summarized in an additional file [Additional file 5: Table S5]. In addition to pMF1.19 and pMF1.20, the pMF1.1- pMF1.5 proteins each had a unique homologue in *M. stipitatus*DSM 14675, from MYSTI_04154 to MYSTI_04158 (YP 007361138.1-YP 007361142.1) (Fig. 6c). It is also noted that, although there is no gene coding for mobility systems [32], and we have not yet observed conjugative transfer of the plasmid between *Myxococcus* strains, the pMF1.2 and its homologue MYSTI_04155 had an AAA_10 and TraC-F-type motifs, both were reported to relate to conjugative transfer [33, 34].

## Conclusions

*M. fulvus* 124B02 is a typical strain of *Myxococcus fulvus**.* The complete genome sequence of *M. fulvus* 124B02 is much larger than the other sequenced genomes of *Myxococcus* strains. The phylogenomic analysis of total genome sequence indicates that *M. fulvus* 124B02 is closer to *M. stipitatus*DSM 14675, rather than *M. xanthus* DK1622 or *M. fulvus* HW-1. Multiple copies of the pMF1.19 and pMF1.20 homologues in different myxobacterial strains suggest that myxobacterial genomes are open, not only to being subject to integrate foreign DNA sequences but also to being duplicated by self, in which the pMF1 plasmid played important roles. The bioinformatics analyses, together with the similar G+C contents of pMF1 and myxobacterial genomes, suggested that pMF1 had a longstanding co-adaption with myxobacteria, probably involving in the expansion of myxobacterial genomes.

## Additional files

Additional file 1: Table S1. Comparison of COG assignments of the genes between *M. fulvus* 124B02 and *M. stipitatus* DSM 14675. (XLSX 11 kb) Additional file 2: Table S2. Comparison of COG assignments of the genes between *M. fulvus 124B02* and *M. fulvus* HW-1. (XLSX 11 kb) Additional file 3: Table S3. Comparison of COG assignments of the genes between *M. fulvus* 124B02 and *M. xanthus* DK1622. (XLSX 11 kb) Additional file 4: Table S4. Strain-specific genes in the family of DNA replication, recombination and repair in *M. fulvus* 124B02, compared with DSM 14675. (XLSX 10 kb) Additional file 5: Table S5. Homologues of pMF1.19 and pMF1.20 in *M. fulvus* 124B02. (XLSX 9 kb)

## Abbreviations

IGD: intra-chromosomal gene duplication
